# 

**Published:** 1903-06

**Authors:** 


### BOOKS RECEIVED.

Veasey's Ophthalmology. A Manual of Diseases of the Eye. For Students and General Practitioners. By Clarence A. Veasey, A. M., M. D., Demonstrator of Ophthalmology in Jefferson Medical College, Philadelphia. 12mo. 410 pages, with 194 engravings and 10 full-page colored plates. Philadelphia and New York: Lea Brothers & Co. 1903. [Cloth, $2.00 net.]

How to Keep Well. An Explanation of Modern Methods of Preventing Disease. By Floyd M. Crandall, M. D. 12mo, pp. 528. New York: Doubleday, Page & Company. 1903.

Transactions of the American Dermatological Association at its Twenty-sixth Annual meeting held at Boston, Mass., September 18-20, 1902. Frank Hugh Montgomery, M. D., Secretary. Chicago: P. F. Pettibone & Co. 1903.

The Practical Medicine Series of Year Books. Ten volumes. Issued under the general editorial charge of Gustavus P. Head, M. D., Professor of Laryngology and Rhinology in the Chicago Post-Graduate Medical School. Vol. V., Obstetrics. Edited by Reuben Peterson, M. D., Professor of Obstetrics and Gynecology, University of Michigan. 12mo, pp. 204. Chicago: The Year Book Publishers. 1903. [Price, $1.25; entire series, $7.50.]

Twenty-sixth Annnal Report of the Michigan State Board of Health. For the fiscal year ending June 30, 1901. Henry B Baker, M. D., Secretary. Lansing: Wynkoop, Hallenbeck, Crawford Co., State Printers. 1902.

The Medical and Surgical Uses of Electricity. Including the X-ray, Finsen Light, Vibratory Therapeutics, and High Frequency Currents. By A. D. Rockwell, A. M., M. D., Formerly Professor of Electro-Therapeutics in the New York Post-Graduate Medical School and Hospital. Octavo, pp. 672. With 250 illustrations. New edition. New York: E. B. Treat & Company. 1903. [Price, $5.00].

The Diagnosis of Diseases of Women. A Treatise for Students and Practitioners. By Palmer Findley, M. D., Instructor in Obstetrics and Gynecology in Rush Medical College, in affiliation with the University of Chicago. In one octavo volume of 494 pages, richly illustrated with 210 engravings and 45 full-page plates in colors and monochrome. Philadelphia and New York: Lea Brothers & Co. 1903. [Cloth, $4.50 net; leather, $5.50 net.]

International Clinics. A Quarterly of Illustrated Clinical Lectures and especially prepared original Articles on Treatment, Medicine, Surgery, Neurology, Pediatrics, Obstetrics, Gynecology, Orthopedies, Pathology, Dermatology Ophthalmology, Otology, Rhinology, Larynology, Hygiene, and other topics of interest to Students and Practitioners, by leading members of the medical profession throughout the world. Edited by A. O. J. Kelly, A.M., M. D., Philadelphia, with the collaboration of Wm. Osler, M. D., Baltimore; John H. Musser, M. D., Philadelphia; Jas. Stewart, M. D., Montreal; John B. Murphy, M. D., Chicago; Thomas M. Rotch, M. D., Boston; John G. Clark, M. D., Philadelphia; James J. Walsh, M. D., New York; J. W. Ballantyne, M. D., Edinburgh; John Harold, M. D., London; Edmund Landolt, M. D., Paris, and Richard Kretz, M. D., Vienna, with regular correspondents in Montreal, London, Paris, Berlin, Vienna, Leipsic, Brussels and Carlsbad. Volume I. Thirteenth series. 1903. Philadelphia: J. B. Lippincott Company. 1903. [Cloth, $2.00.]

